# Distressed personality is associated with late adverse effects in long-term survivors of Hodgkin lymphoma

**DOI:** 10.2340/1651-226X.2024.40312

**Published:** 2024-08-04

**Authors:** Alv A. Dahl, Knut B. Smeland, Siri Eikeland, Unn-Merete Fagerli, Hanne S. Bersvendsen, Alexander Fosså, Cecilie E. Kiserud

**Affiliations:** a National Advisory Unit for Late Effects after Cancer Therapy, Oslo University Hospital, Oslo, Norway; b Department of Oncology, Oslo University Hospital, Oslo, Norway; c Department of Oncology, St Olav’s Hospital, Trondheim, Norway; d Institute of Clinical and Molecular Medicine, NTNU, Trondheim, Norway; e Department of Oncology, University Hospital of Northern Norway, Tromsø, Norway; f KG Jebsen Centre for B-cell malignancies, University of Oslo, Oslo, Norway

**Keywords:** Hodgkin lymphoma, Cancer survivors, type D personality, Late adverse effects

## Abstract

**Background and purpose:**

There are few studies of personality traits in long-term Hodgkin lymphoma survivors (HLSs) treated according to contemporary stage-and risk-adapted approaches. The Distressed Personality (DP) Scale covers negative affectivity and social inhibition. We examined differences in self-reported late adverse effects (LAEs) between HLSs with and without DP and other explanatory variables.

**Material and methods:**

This cross-sectional questionnaire-based study included a population-based cohort of HLSs treated from 1997 to 2006, aged 8–49 years at diagnosis, and alive in 2016. Among 518 eligible HLSs, 303 responded (58%), and 294 completed the DP scale. DP was defined by scores above cut-off on both the negative affectivity and social inhibition subscales. LAEs studied were major depression, posttraumatic stress disorder, sleep problems, obesity, neuropathy, fatigue, memory problems, and general health. DP and 10 other explanatory variables were tested against LAEs as dependent variables in multivariable regression analyses.

**Results:**

The mean age at survey was 45.9 years (standard deviation [SD] 4.6), mean follow-up time 16.7 years (SD 3.0), and 48% were females. Eighty-two HLSs had DP (28%, 95% confidence interval 23% – 33%). All LAEs except obesity were significantly more common/had higher mean score in HLSs with DP. In multivariable analyses, presence of DP was significantly associated with all LAEs except obesity.

**Interpretation:**

The presence of DP is common among HLSs. The presence of DP was associated with most self-report LAEs examined. Including assessment of personality traits in the survivorship care plans of HLSs should be considered. Prospective studies assessing the influence of pretreatment DP on LAEs are warranted.

## Introduction

Since the mid-1970s psychosocial concepts such as quality of life, mental distress, and fatigue in cancer survivors have become familiar to the oncological community [1]. Less research has concerned the relevance of basic personality traits for survivorship problems. The Five Factor Model (FFM) for such traits is currently the most accepted model [2] and contains the following traits: neuroticism, also called negative affectivity (i.e. the tendency to experience negative emotions under stress); extraversion (i.e. ability to be social, assertive, and active); openness to new experiences (i.e. ability to be curious and creative); agreeableness (i.e. ability to be friendly, easygoing, and collaborative); and conscientiousness (i.e. ability to be reliable, organized, and persistent).

Basic personality traits are determined by heredity and environment and they are firmly established during adolescence. Thereafter, such traits remain stable, but can be modified during the rest of the life span. Recently, the influence of stress, like cancer, on personality traits has been documented [2]. In addition, it has appeared that such traits can be modified by targeted interventions [3].

Among basic personality traits neuroticism has been mostly studied in long-term cancer survivors. Increased neuroticism is regularly associated with more late adverse effects (LAEs), increased mental distress, and reduced quality of life [4]. Individuals who score highly on both neuroticism and on social inhibition (low extraversion), are considered to have Distressed Personality (DP) [5]. The presence of DP in cancer survivors is associated with increased comorbidity burden, health care utilization, as well as inferior quality of life, and mental distress [6, 7]. To our knowledge, health problems associated with DP have only been studied in more detail in survivors of colorectal cancer [8, 9]. The presence of DP was significantly associated with lower quality of life and poorer disease-specific health status, as well as less physical activity compared to survivors without DP.

Hodgkin lymphoma (HL) mostly affects young adults and has an excellent long-term prognosis [10], but with high risk for LAEs [11] such as fatigue [12, 13], second cancer [14, 15], cardiovascular diseases [14, 15], and peripheral neuropathy [16]. The impact of personality traits on LAEs has so far not been studied among HL survivors (HLSs). Therefore, we wanted to examine whether HLSs with DP more frequently reported common LAEs than HLSs without DP. Our hypotheses were that the presence of DP in HLSs was significantly associated with increased burden of LAEs, and that DP was significantly associated with most LAEs when compared to other explanatory variables.

## Material and methods

The current cross-sectional study of HLSs after contemporary stage and risk adapted treatment approaches was based on questionnaire data covering socio-demographic, psychological, and lifestyle characteristics, as well as clinical data from the time of diagnosis and treatment of HL from patients’ charts [13].

### Sample characteristics

This population-based study from three health regions in Norway, concerned HLSs treated from 1997 to 2006, aged 8–49 years at diagnosis, and alive at the end of 2016 as identified by the Norwegian Cancer Registry. Patients were treated by contemporary stage- and risk-adapted strategies, that is, 2–4 cycles of a combination of doxorubicin, dacarbazine, vinblastine, and bleomycin (ABVD) followed by involved-site radiotherapy for limited stage and 6–8 cycles of ABVD or a combination of doxorubicin, cyclofosfamide, etoposide, vincristine, bleomycin, procarbazine, and prednisolone (BEACOPP) (for high risk patients from 1999) for advanced stage, as described in Table 1 and in previous reports [13, 16]. Among 518 eligible HLSs, 303 responded (58% response rate), and 294 completed the DS-14 scale. An attrition analysis showed that non-respondents were younger at diagnosis and survey, and they were more frequently men [13].

**Table 1:** Characteristics of all respondents and in those with or without DP (*N* = 294).

Variables	DP present (*N* = 82)	DP absent (*N* = 212)	*p*-value	Total sample (*N* = 294)
*Age at diagnosis, mean (SD)*	28.3 (9.7)	29.4 (9.4)	0.38	29.1 (9.5)
*Age at survey, mean (SD)*	44.8 (9.4)	46.3 (9.7)	0.24	45.9 (9.7)
*Follow-up time, mean (SD)*	16.5 (3.1)	16.8 (2.9)	0.35	16.7 (3.0)
*Histology, N (%)*			0.58	
* *Classical HL	75 (92)	189 (89)		264 (90)
NLPHL	6 (7)	22 (10)		28 (9)
Unclassified	1 (1)	1 (1)		2 (1)
*Stages, N (%)*			0.75	
I–IIA	49 (60)	131 (62)		180 (61)
IIB–IV	33 (40)	81 (38)		114 (39)
*B-symptoms at diagnosis, N (%)*	31 (38)	66 (31)	0.47	97 (33)
*Treatment modalities, N (%)*				
Antracyclines	77 (94)	199 (94)	0.99	276 (94)
Chemotherapy	77 (94)	200 (94)	0.89	277 (94)
ABVD	63 (77)	168 (79)	0.65	231 (79)
BEACOPP	6 (7)	15 (7)	0.95	21 (7)
HDT-ACST	11 (13)	26 (12)	0.79	37 (13)
Radiotherapy	64 (78)	163 (77)	0.83	227 (77)
*Second cancer, N (%)*	5 (6)	18 (9)	0.49	23 (8)

SD: standard deviation; DP: distressed personality; HL: Hodgkin lymphoma; NLPHL: Nodular lymphocyte predominant Hodgkin’s lympho; ABVD: combination of doxorubicin, dacarbazine, vinblastine, and bleomycin; BEACOPP: combination of doxorubicin, cyclofosfamide, etoposide, vincristine, bleomycin, procarbazine, and prednisolone; HDT-ACST: high-dose chemotherapy and autologous stem-cell transplantation.

### Main outcome measure

*The Distressed Personality Questionnaire (DS14)* is, a 14-item questionnaire that examines negative affectivity and social inhibition, with seven items covering both these personality traits [5]. Each item of the DS14 is scored from 0 (False) to 4 (True), giving sum scores on each trait from 0 to 28. DP is defined by a sum score of ≥ 10 on each trait [5]. The internal consistencies measured by Cronbach’s coefficient alpha were 0.89 for both traits.

### Self-rated measures

*Fatigue Questionnaire (FQ)* consists of two subscales for physical and mental fatigue that are added as total fatigue score. Each item is rated from 0 (less than before/not at all) to 3 (much more than usual), with a possible total fatigue score ranged from 0 to 33, with higher score implying more fatigue [17, 18]. Alpha for total fatigue was 0.93.

The *Impact of Event Scale (IES-6)* assesses post-traumatic stress symptoms related to the HL trajectory with two items relating to intrusion, avoidance, and hyperarousal, respectively. Each item is rated from 0 (not at all) to 4 (very much), providing a total severity score from 0 to 24. A probable case of post-traumatic stress disorder (PTSD) was defined by a sum score ≥ 9 [19]. Alpha was 0.89.

*Patient Health Questionnaire-9 (PHQ-9)* assesses depressive symptoms experienced during the last 2 weeks, where each item is scored from 0 (not at all) to 3 (nearly every day), providing a severity score ranging from 0 to 27. A case of probable major depressive episode (MDE) was defined by a sum score ≥ 10 [18, 20]. Alpha was 0.87.

*Metamemory Questionnaire (MMQ)* covers memory problems over the last week with nine items intended to capture memory performance by their summary score. Two items cover general memory, three concern semantic memory, and four relate to working memory. Each item is scored from 0 (no/never) to 2 (yes a lot/often), with a summary score ranging from 0 to 18, and higher score implying more severe memory deficit [21]. Alpha was 0.88.

*EORTC QLQ-CIPN20* is a 20-item questionnaire assessing neuropathic symptoms. The neuropathy sum score is based on 18 items, excluding questions regarding pedal use while driving and erectile dysfunction in men. The severity is scored from 1 (not at all) to 4 (very much), and the scores are transformed into 0–100 scales. Higher scores indicate more neuropathy [22, 23]. Alpha was 0.89.

*Short Form 36 (SF-36) General health* was rated by the SF-36 subscale consisting of five items. Item scores were transformed to 0 (worst) to 100 (best) scores based on the SF-36 algorithm. Alpha was 0.83 [24].

### Other variables

#### Socio-demographic variables

Partner relationship was dichotomized as either married/cohabiting or not living with a partner. Short education was defined as ≤ 12 years versus long education > 12 years. Income status was dichotomized into paid work versus pensions/economic support.

#### Oncological variables

Data on histology, stage, and treatment were extracted from medical files and from the lymphoma database for the patients treated at Oslo University Hospital.

#### Comorbidity variables

Cardiovascular diseases were self-reported and included myocardial infarction, angina pectoris, cardiac failure, other cardiac diseases, diabetes, and stroke. The presence of any cardiovascular disease was categorised as ‘yes’ or ‘no’. Other somatic diseases were also reported with a modification of the Self-administered Comorbidity Questionnaire [25] and concerned gastric ulcer, arthritis, arthrosis, kidney-, liver -, chronic pulmonary-, and thyroid diseases. The presence of each somatic disease was categorised as ‘no’ or ‘yes’. The number of self-reported somatic diseases was categorized as none, one, or ≥ 2.

### Health and lifestyle variables

Dental health was assessed as ‘good’ or ‘poor’. Sleep problems were present if either insomnia or early awakening without going back to sleep were reported several times a week for the last 3 months. Daily smoking meant current smoking of any number of cigarettes. Obesity at survey was defined as body mass index ≥ 30 kg/m^2^. Daily physical activity *≥* 30 min was scored as ‘yes’ or ‘no’. Intake of alcohol was dichotomized as any amount of alcohol consumed ≥ once a week or less frequently.

### Statistical analyses

Continuous variables were presented as means and standard deviation (SD), categorical variables as numbers and rates. Descriptive statistics were performed with chi-square tests for categorical variables and independent sample t-tests for continuous variables, in case of skewed distributions Mann–Whitney U-tests were performed. Between-group differences on continuous variables were also expressed as effect sizes with Cohen’s coefficient *d.* Internal consistencies of scales were calculated by Cronbach’s coefficient alpha.

Multivariable logistic and linear regression analyses were used to investigate associations between eight self-reported LAEs (neuropathy, total fatigue score, memory problems, probable MDE, probable PTSD, sleep problems, obesity, and general health) as dependent variables versus 10 selected explanatory variables (presence of DP, age at survey, stage of HL, follow-up time, B-symptoms at diagnosis, sex, level of education, partner status, income status, cardiovascular disease, and somatic comorbidity). The explanatory variables were tested for multicollinearity and none was observed. The strength of associations was expressed as odds ratios (ORs) with 95% confidence intervals (95% CIs) for logistic regression and as B and standardized betas for linear regression analyses. Associations with *p*-values < 0.05 were considered statistically significant, and all tests were two-sided. All analyses were performed using IBM SPSS statistics version 28 (Armonk, NY).

## Results

### Characteristics of the total sample

The mean age of the sample at diagnosis was 29.2 years (SD 9.5), at survey 45.9 years (SD 9.6), and the mean follow-up time was 16.7 years (SD 3.0) (Table 1). Ninety percent had histology of classical HL, 61% had stage I-IIA, and 33% had B-symptoms at diagnosis. Close to all HLSs had received chemotherapy (94%) and the majority had received radiotherapy (77%). Thirteen per cent had undergone high-dose chemotherapy with autologous stem transplant due to progression or relapse.

The sample consisted of 48% females and 80% were in partnered relationships (Table 2). Concerning income status 70% held paid work, 17% were on disability pension, and 13% received other types of economical support from the welfare system.

**Table 2:** Findings of the DP present and absent groups and the total sample (*N* = 294).

Variables	DP present (*N* = 82)	DP absent (*N* = 212)	*p*-value	Effect sizes^1^	Total sample (*N* = 294)
*Sex, N (%)*			0.72		
Female	41 (50)	101 (48)			142 (48)
Male	41 (50)	111 (52)			152 (52)
*Partnered relationship, N (%)*	58 (71)	178 (84)	**0.01**		236 (80)
*Level of education, N (%)*			**< 0.001**		
Short (< 12 years)	56 (68)	75 (35)			131 (45)
Long (≥ 12 years)	26 (32)	137 (65)			163 (55)
*Current income status, N (%)*			**< 0.001**		
Paid work	45 (55)	160 (76)			205 (70)
On pensions/support	37 (45)	52 (24)			89 (30)
*Neuropathy, mean (SD)*	21.2 (16.8)	9.7 (10.8)	**< 0.001**	0.90	12,9 (13.7)
*Cardiovacular disease, N (%)*	21 (26)	58 (27)	0.76		79 (27)
*Other somatic diseases, N (%)*			0.11		
None	38 (46)	86 (40)			124 (42)
One	22 (27)	84 (40)			106 (36)
≥ Two	22 (27)	42 (20)			64 (22)
*General health, mean (SD)*	43.6 (23.9)	67.4 (25.7)	**< 0.001**	−0.94	60.7 (27.4)
*Poor dental health, N (%)*	14 (15)	28 (14)	0.36		42 (15)
*Cases of depression, N (%)*	40 (49)	27 (13)	**< 0.001**		67 (23)
*Cases of PTSD, N (%)*	46 (59)	37 (19)	**< 0.001**		83 (30)
*Total fatigue, mean (SD)*	18.9 (5.7)	14.3 (5.5)	**< 0.001**	0.83	15.6 (5.9)
*Metamemory score, mean (SD)*	8.8 (4.3)	5.7 (3.7)	**< 0.001**	0.81	6.6 (4.2)
*Sleep problems, N (%)*	36 (44)	48 (22)	**< 0.001**		82 (28)
*Obesity (BMI ≥ 30kg/m^2^), N (%)*	23 (28)	42 (20)	0.11		65 (22)
*Daily smoking, N (%)*	18 (22)	28 (13)	0.06		46 (16)
*Alcohol ≥ once a week, N (%)*	24 (30)	96 (46)	**0.01**		120 (41)
*< 30 min physical activity, N (%)*	29 (36)	73 (35)	0.81		102 (35)

1 Effect sizes (Cohen’s *d*) ≥ 0.80 indicate differences of great clinical significance.

SD: standard deviation; DP: distressed personality; PTSD: post-traumatic stress disorder; BMI: body mass index.

p-values < 0.05 were considered significant (shown in bold)

### Rates and means of DP and subscales

Among HLSs 82 had DP (28%, 95% CI: 23%–33%) and for them the sex distribution was equal. The rate of negative affectivity was 38% (95% CI: 33%–44%) and of social inhibition 43% (95% CI: 37%–49%) also with no significant sex differences. The mean score of negative affectivity was 7.6 (SD 5.4) in male HLSs and 9.1 (SD 5.4) in females (*p* = 0.016), and 8.3 (SD 5.5) in the total sample. The corresponding mean scores for social inhibition were 9.4 (SD 5.9) in males, 9.6 (SD 5.7) in females (*p* = 0.75), and 9.5 (SD 5.8) in total HLSs sample.

### Comparisons of HLSs with and without DP

At survey, HLSs with DP more frequently had short education and were more likely to live without a partner as well as to receive pensions or other economic welfare support. Rates of probable cases of MDE and PTSD, and sleep problems were also higher among HLSs with DP, who also reported higher symptom burden of peripheral neuropathy, fatigue and memory problems, and lower level of general health compared to those without DP (Table 2).

### Multivariable regression analyses of LAEs

In the multivariable logistic analyses of LAEs defined categorically (Table 3), the presence of DP was positively associated with probable MDE and PTSD, and sleep problems, but not with obesity. Being on pensions/economic support was significantly associated with probable MDE and PTSD. Other explanatory variables less frequently showed significant associations with these outcome variables, and HL stages, presence of B-symptoms, short education, and no partnered relationship showed none.

**Table 3:** Multivariable logistic regression analyses of explanatory variables and health problems at survey as dependent variables.

Variables	Probable MDE			Probable PTSD			Sleep problems			Obesity		
	OR	95% CI	*p*-value	OR	95% CI	*p*-value	OR	95% CI	*p*-value	OR	95% CI	*p*-value
*DP present*	6.48	3.17 – 13.25	**< 0.001**	5.63	2.93 – 10.84	**< 0.001**	2.18	1.18 – 4.03	**0.013**	1.28	0.63 – 2.33	0.57
*Age at survey*	1.00	0.96 – 1.04	0.92	1.00	0.97 – 1.03	0.76	1.00	0.97 – 1.03	0.94	1.02	0.98 – 1.05	0.41
*Female sex*	2.32	1.16 – 4.64	**0.017**	1.81	0.96 – 3.41	0.07	1.45	0.81 – 2.58	0.21	1.04	0.57 – 1.90	0.57
*Stage IIB-IV*	1.22	0.68 – 2.56	0.42	1.25	0.55 – 2.85	0.60	1.47	0.83 – 2.61	0.19	0.84	0.45 – 1.56	0.84
*B-symptoms at diagnosis*	1.07	0.95 – 1.20	0.30	1.28	0.54 – 3.02	0.57	0.97	0.78 – 1.20	0.76	0.96	0.76 – 1.30	0.81
*Follow-up time*	0.89	0.8 – 0.99	**0.047**	1.00	0.90 – 1.10	0.95	1.02	0.93 – 1.12	0.69	0.91	0.83 – 1.01	0.07
*Somatic comorbidity*			0.09			0.12			0.52			0.40
None (reference)	1.00	-	-	1.00	-	-	1.00	-	-	1.00	-	-
1 disease	1.93	0.88 – 4.25	0.10	1.08	0.53 – 2.20	0.83	1.14	0.60 – 2.17	0.68	0.65	0.33 – 1.29	0.15
≥ 2 diseases	2.59	1.08 – 6.22	**0.034**	2.20	0.99 – 4.89	0.05	1.52	0.74 – 3.15	0.26	1.01	0.48 – 2.16	0.97
*Cardiovascular disease*	1.37	0.65 – 2.90	0.41	1.30	0.66 – 2.57	0.45	1.91	1.04 – 3.54	**0.037**	1.74	0.92 – 3.28	0.09
*Short education*	1.05	0.52 – 2.13	0.89	1.33	0.70 – 3.52	0.38	1.59	0.88 – 2.86	0.12	1.87	1.01 – 3.47	**0.045**
*No partner*	1.68	0.80 – 3.54	0.18	0.84	0.40 – 1.78	0.65	1.78	0.92 – 3.43	0.09	1.53	0.76 – 3.08	0.24
*On pension/support*	3.15	1.59 – 6.24	**0.001**	2.48	1.29 – 4.76	**0.007**	1.43	0.79 – 2.61	0.24	1.00	0.98 – 1.04	0.99

MDE: major depressive episode; PTSD: posttraumatic stress disorder; OR: odds ratio; CI: confidence Interval; DP: distressed personality.

p-values < 0.05 were considered significant (shown in bold)

In the multivariate linear regression analyses of the four LAEs measured dimensionally, the presence of DP was positively associated with all of them: neuropathy, total fatigue, memory problems, and poorer general health (Table 4). Associations were also observed for the presence of B-symptoms at diagnosis and being on pensions/economic support. Being female and having ≥2 comorbid diseases were positively associated with three of these LAEs. The other explanatory variables showed fewer significant associations with these LAEs and follow-up time showed none.

**Table 4:** Multivariable linear regression analyses of explanatory variables and health problems survey as dependent variables.

Variables	Neuropathy			Total fatigue			Memory problems			General health		
	B	beta	*p*-value	B	beta	*p*-value	B	beta	*p*-value	B	beta	*p*-value
*DP present*	9.33	0.31	**< 0.001**	3.89	0.30	**< 0.001**	2.84	0.31	**< 0.001**	−19.60	−0.32	**< 0.001**
*Age at survey*	0.09	0.06	0.24	0.001	0.001	0.98	0.03	0.07	0.26	0.02	0.01	0.87
*Female sex*	2.74	0.10	**0.047**	1.77	0.15	**0.005**	1.33	0.16	**0.004**	−3.96	−0.07	0.13
*Stage IIB-IV*	−1.10	−0.04	0.42	0.33	0.03	0.59	−0.02	−0.002	0.97	1.03	0.02	0.69
*B-symptoms at diagnosis*	0.33	0.14	**0.005**	0.16	0.15	**0.003**	0.08	0.11	**0.042**	−0.66	−0.14	**0.003**
*Follow-up time*	0.06	0.01	0.79	−0.02	−0.01	0.88	−0.03	−0.02	0.74	−0.58	−0.06	0.18
*Somatic comorbidity*												
None (reference)	-	-	-	-	-	-	-	-	-	-	-	-
1 disease	2.19	0.08	0.10	1.80	0.15	**0.009**	0.99	0.12	0.052	−2.74	−0.05	0.35
≥ 2 diseases	9.02	0.27	**< 0.001**	2.26	0.16	**0.006**	0.64	0.06	0.30	−15.71	−0.24	**< 0.001**
*Cardiovascular disease*	2.58	0.08	0.09	1.44	0.11	**0.038**	0.39	0.04	0.45	−8.64	−0.14	**0.003**
*Short education*	3.72	0.14	**0.009**	0.44	0.04	0.49	0.92	0.11	0.054	0.26	0.01	0.93
*No partner*	−2.30	−0.07	0.17	−0.46	−0.03	0.54	−0.97	−0.09	0.09	1.96	0.03	0.54
*On pension/support*	5.82	0.20	**< 0.001**	3.88	0.30	**< 0.001**	1.37	0.15	**0.008**	−20.72	−0.35	**< 0.001**

DP: distressed personality

p-values < 0.05 were considered significant (shown in bold)

## Discussion

In this population-based survey, 28% of HLSs had DP. These HLSs had significantly higher rates of probable MDE and PTSD, and sleep problems compared to HLSs without DP as well as significantly higher mean scores on peripheral neuropathy, fatigue, and memory problems, and lower mean score on general health. In the multivariable analyses the presence of DP was significantly associated with all health problems examined, except for obesity.

The prevalence of DP in our HLSs cohort was within the upper range observed in a randomly selected and age- and gender-stratified Norwegian population sample [26]. Here 24% (95% CI: 20%–28%) of the participants were found to have DP. In a review of 19 studies with samples from the general populations of several countries, the prevalence of DP varied between 17% and 39%, indicating sampling and cultural variations of the DP prevalence [27]. In the latter review, the presence of DP was significantly associated with more depression and anxiety, and lower health status, as also found in this study of HLSs.

In a Dutch study of 3,080 cancer survivors, the overall DP prevalence was 19% (95% CI: 17%–20%) in the total sample [6]. This overall prevalence of DP for cancer survivors is lower than that observed in our HLSs sample, despite their inclusion of 28% HLSs, but the prevalences for separate cancer types were not given. Like in our study, they reported significantly lower mean score on general health, and higher rates of probable depression and anxiety in survivors with DP compared to survivors without [7]. The same research group also found a prevalence of DP of 21% (95% CI: 19%–22%) in long-term survivors of colorectal cancer [9]. In the latter study, survivors with DP also drank less alcohol, but differed significantly from those without DP concerning obesity and smoking, which is different from the observations made in our study. These findings should be considered in the light of differences in type of cancer, sampling, health care including treatment, and culture.

Cardiovascular diseases are common in HLSs [14, 15, 28], and the rate of such problems was 27% in our sample based on self-report. There was no difference in the rate of cardiovascular diseases between HLSs with or without DP. However, in studies of both general populations and different patient groups, there is a considerable documentation of more cardiovascular conditions in individuals with DP [5]. Physical activity is recommended as part of a healthy lifestyle for all, and especially as prophylaxis of cardiovascular diseases in HLSs [29]. However, high neuroticism, like in DP, is associated with less physical activity in both Norwegian [30] and French [31] population samples.

We included disease- and treatment-related variables as possible confounders in our multivariable models. Since most HLSs received chemotherapy (94%) and/or radiotherapy (77%), these factors are not fit as explanatory variables, and the same is true regarding HL histology. We therefore included stage of HL and presence of B-symptoms at diagnosis as HL-related explanatory variables, together with follow-up time. Stage and follow-up time were not associated with LAEs, except for probable MDE being associated with the latter variable. However, B-symptoms were positively associated with neuropathy, fatigue, and memory problems and negatively associated with general health. Compared to the presence of DP and being pensioned/on economic support, the disease- and treatment-related variables were less relevant for health problems in HLSs.

Our results support the hypothesis that having DP is significantly associated with self-report of a wide range of LAEs in HLSs. This result confirms the relevance of personality traits particularly neuroticism (negative affectivity), for LAEs as observed in several other types of cancer survivors [4]. As stated in the introduction, recent research has shown that considerable modification of personality traits can be the result of both stressful life events and systematic interventions [2, 3, 32]. From our cross-sectional analysis, we can only speculate whether the two traits examined with DP (negative affectivity and social inhibition) modify the perception of common LAEs in HLSs or that the HL trajectory aggravate these two personality traits. These are interesting perspective as LAEs per se vary as to their response to treatment. There are effective treatments for several common LAEs in HLSs [4]. However, less is documented for memory problems, even though, there is some progress regarding effects of cognitive training on functioning in cancer survivors [33]. Personality traits can change by self-help, psychological interventions, and psychopharmacology [3, 32]. Clinicians who care for HLSs may, therefore, take interest in the personality traits of HLSs with multiple LAEs, and eventually consider possible interventions. Including a short screening tool for personality traits such as the DS14 could be helpful in this regard, and they should be considered for survivorship care designs. The DS14 has been used in many clinical studies mostly within cardiology. It has however also been criticized [34], mainly for the combination of two independent FFM traits: neuroticism and extraversion. The definition of DP includes both high neuroticism (i.e. low stress tolerance) and low extraversion (i.e. low interest in social interaction) as they both are associated with health problems [5].

Strengths of our study are the considerable sample size of HLSs participating in a population-based survey more than a decade after diagnosis and use of established instruments with good psychometric properties. One limitation is eventual responder bias since younger and male HLSs was over-represented among non-responders implicating that our responders may have more biopsychosocial problems. Another limitation is the cross-sectional study design, which only allows for statements about association and not about causality, only associations. Prospective studies are warranted to explore whether an individual’s levels of negative affectivity and/or social inhibition change because of the HL diagnosis and treatment and how pre-therapeutic DP may influence the development of other LAEs. Another limitation is the lack of normative Norwegian data on the DS14.

The prevalence of DP among HLSs was at the upper end of the range of various normative samples and possibly higher than reported in other samples of long-term cancer survivors. Multivariable analyses showed that several LAEs in HLSs were significantly associated with the presence of DP. Oncologist and other health care providers caring for HLSs should consider the use of a screening test for personality traits in their care plans.

## Data Availability

According to Norwegian data legislation, the data of this study cannot be made generally available. Requests may be made to the senior author.
